# The Epilepsy Risk Awareness (ERA) Scale: A New Era for Holistic Risk Assessment in Epilepsy

**DOI:** 10.3389/fneur.2020.00465

**Published:** 2020-06-11

**Authors:** Rachel Ison, Virad Kisan, Christine Cole, Heather Angus-Leppan

**Affiliations:** ^1^Clinical Neurosciences, Royal Free London NHS Foundation Trust, London, United Kingdom; ^2^University College London Medical School, London, United Kingdom; ^3^Harrow Neurology Specialist Nursing Service, Middlesex, United Kingdom; ^4^UCL Queen Square Institute of Neurology, London, United Kingdom

**Keywords:** epilepsy, risk awareness, risk assessment, clinical scale, sudden unexpected death in epilepsy, holistic, quality of life

## Abstract

**Objective:** Best care in epilepsy balances protecting people with epilepsy from risks and avoiding undue restrictions in order to improve quality of life. To date, no single risk assessment tool has been widely adopted by both people with epilepsy and health-care professionals such as specialist epilepsy nurses. The present research refined the Epilepsy Risk Awareness (ERA) Scale, a validated and holistic risk assessment tool, by assessing test–retest reliability of each question and incorporating suggestions from patients regarding design and content.

**Methods:** The draft clinical scale was administered to 102 adult participants from the Epilepsy Service at the Royal Free London National Health Service (NHS) Foundation Trust on two occasions. Quantitative and qualitative analyses were conducted—intraclass correlation coefficient (ICC) estimates were used to assess test–retest reliability of questions, and thematic analysis was used to analyze participants' comments and feedback. Following analysis, the ERA Scale was amended. Of the 102 participants, 32 conducted a further review of the revised ERA Scale to test completion time and provide final comments.

**Results:** ICC reliability level estimates varied from “poor to moderate” to “good to excellent,” and four qualitative themes were identified. The ERA Scale was amended accordingly to enhance practicality and usefulness, reducing completion time to approximately 5 min.

**Significance:** The ERA Scale is a validated tool that aims to change clinical practice by standardizing risk assessment in epilepsy, providing a holistic approach that focuses on improved safety and quality of life.

## Introduction

Health-care professionals face the challenge of helping people with epilepsy live not only longer but also better. Epilepsy is one of the most common neurological conditions (1) with associated medical, environmental, psychological, and social consequences that may profoundly affect an individual's life. An act as simple as taking a bath can potentially be fatal, and issues surrounding employment (2), social isolation (3), and stigma (4) are well documented. As seizure recurrence is unpredictable and fluctuating, there is an ongoing risk for people with epilepsy (3). Therefore, while accurate diagnosis and treatment are vital, it is also essential to consider individuals' lives holistically when assessing risk. No single tool has been widely adopted by people with epilepsy and health-care professionals, such as specialist epilepsy nurses, to standardize and unify risk assessment in epilepsy.

Patient-centered care involves a shift from patients being passive recipients to active agents in their health care. Self-management and active involvement enable informed decisions (5) and support psychological adjustment to chronic conditions (6), in addition to improving outcomes (7), feelings of empowerment and control (8), and quality of life (9). Monitoring a health condition with tools such as scales is a form of self-management (10) that is of proven value in neurology (11).

A small range of risk-related assessment tools exist for people with epilepsy. For example, in order to prevent sudden unexpected death in epilepsy (SUDEP), the SUDEP and Seizure Safety Checklist that became the smartphone-based app Epilepsy Self-Monitor (EpSMon) (12) allows people with epilepsy to monitor their condition and assess their risk while providing education accordingly. Some scales assess how often people with epilepsy engage in self-management practices, such as the Epilepsy Self-Management Scale (13), and the Chalfont Seizure Severity Scale (14) assesses seizure severity. Charities such as the Epilepsy Society have their own risk assessment templates on their websites (15). Additionally, there are scales that measure quality of life in epilepsy, for example, QOLIE-10 (16). Even though each of these tools has a specific focus area, there is not currently one tool that brings all of these elements together.

The Epilepsy Risk Awareness (ERA) Scale, previously piloted as the ERA Checklist (17, 18), aims to fill this gap, as it was designed to balance protecting people with epilepsy from risks and avoiding undue restrictions, thereby improving quality of life. The ERA Scale has been developed iteratively, and the methodology has incorporated patient and health-care professional involvement throughout (17, 18), It was initially designed as a tool for specialist nurses to use with people with epilepsy and intellectual disabilities (17), but it has evolved owing to clinical need to be used by all people with epilepsy and all health-care professionals. The ERA Scale can be deployed in a clinical or personal setting to assess both immediate risk and longitudinal changes, which can be utilized for clinical and research purposes. This is essential for the successful long-term management of epilepsy, as making changes requires a baseline assessment, given that the condition's trajectory and individual life experiences alter.

The ERA Scale is designed to facilitate more structured, tailored, and sensitive conversations between people with epilepsy and their health-care team, for example, by asking about a need for mental health support. This is particularly important, considering there is a higher prevalence of depression and anxiety in people with epilepsy (1, 19). These more holistic conversations around risk can form the basis of the “agreed and comprehensive written epilepsy care plans” (20) that the National Institute for Health and Care Excellence (NICE) (2013) recommend for all adults with epilepsy, which could improve the communication and information sharing between different health-care teams and services.

The present research assessed test–retest reliability of each question on the ERA Scale and incorporated suggestions from patients regarding its design and content, intending to refine the ERA Scale and reduce completion time from 20 to less than 10 min.

## Methods

### Participants

Participants consisted of 102 consenting adults (18+ years old) with epilepsy [as defined by the 2005 guidelines (21)], who were recruited from the Epilepsy Service at the Royal Free London National Health Service (NHS) Foundation Trust from April to May 2019. The only inclusion criteria were being a consenting adult and having a diagnosis of epilepsy; there were no additional exclusion criteria. Of the 102 participants, there were 56 females and 46 males, ages ranged from 18 to 81 years, and the median age was 43 years. Seven participants have a diagnosis of an intellectual disability.

### Measure

The ERA Scale (version 2, see Supplementary Materials) is a questionnaire designed to assess the risk level of people with epilepsy (17, 18). It is composed of four parts: (A) General Information (10 items); (B) the Chalfont Seizure Severity Scale (14) (10 items and included as an additional measure of the impact of epilepsy); (C) the ERA Scale (the main part of the questionnaire that consists of 48 items); and (D) the Epilepsy Self-Management Scale (13) (38 items, which is optional for participants to complete, included as a comparative measure).

Each part of the questionnaire contains different response options, with Part C (the ERA Scale) allowing participants to select “yes,” “no,” or “not applicable” for each question. At the end of Part C (the ERA Scale) was an optional text box for participants to provide any comments or feedback.

### Procedure

Participants were recruited in person and via phone by the research assistant. Some were approached in person while waiting for an outpatient appointment at the Royal Free Hospital, and the majority were contacted via phone, going alphabetically through the Royal Free London's Epilepsy Database. Participants were provided with written and verbal information about the study, in line with the ethics approval. Informed consent was obtained prior to participants' involvement in the research, either from participants themselves or on their behalf by their families and/or carers if they lacked capacity.

Sample size requirements were calculated based on a test–retest reliability study using intraclass correlation coefficient (ICC) for analysis, which recommends a minimum of 30 heterogeneous samples (22). In order to ensure a substantial sample size of 100, many more were contacted, as it was estimated that half of those contacted would agree to participate and not all participants would complete both phases of the test–retest. In total, 276 patients were contacted, 113 agreed to take part, and 102 participants completed the questionnaire at two points in time. We aimed for a test–retest period of 2 weeks, with no less than 5 days between completion dates. Participants had a choice of how to complete the questionnaire, in person at the outpatient clinic, over the phone, or via an online link to a Google Form that was emailed to participants with an individual study participant number. Participants were permitted to use the same or different method to complete the questionnaire the first and second times, and participants who had not completed it were reminded to via either email or phone call.

Participants were given the time they needed to complete it over the phone. Questions were read to the participants, and their verbatim responses were written down by the research assistant, including when providing comments and feedback in the optional text box at the end of the ERA Scale. Consistency was maintained, as one trained research assistant was responsible for recruitment and data collection and conducted all of the phone calls. Participants could use support from carers and/or family members to complete the questionnaire or were able to have their carers and/or family members complete it on their behalf.

Following the analysis, the ERA Scale (version 2) was amended, producing the final ERA Scale (version 3). In order to conduct a final review of the ERA Scale (version 3), the research assistant contacted the original 102 participants, and 32 responded within the set time period of 2 weeks. All participants opted to complete it via the online link. This final review was to test the time taken for completion and to provide additional comments about the design and content in a feedback box at the end of the questionnaire, as in the previous version.

### Data Analysis

A mixed methods approach was used to gain a more in-depth understanding of the strengths and weaknesses of the ERA Scale (version 2), from both a test–retest reliability and a user experience perspective.

#### Quantitative Analysis

Descriptive statistics were generated for Part A (General Information) and Part B [the Chalfont Seizure Severity Scale (14)] of the ERA Scale (version 2), which summarized demographic information and details of seizures. To assess test–retest reliability of each question in Part C (the ERA Scale) and Part D [the Epilepsy Self-Management Scale (13)], intraclass correlation coefficient (ICC) estimates and their 95% confident intervals were calculated based on a mean rating, absolute agreement, two-way mixed-effects model. Reliability levels of each question were determined based on the criteria outlined by Koo and Li (2016) (22): “ICC values less than 0.5 are indicative of poor reliability, values between 0.5 and 0.75 indicate moderate reliability, values between 0.75 and 0.9 indicate good reliability, and values greater than 0.9 indicate excellent reliability.” Prior to analysis and storage, names were replaced with codes, so participants remained anonymous. Analyses were performed using SPSS version 23.

#### Qualitative Analysis

Thematic analysis was used to analyze participants' comments and feedback, following the steps outlined by Braun and Clarke (23), and data were managed in Microsoft Excel. Based on the nature of the research question and data, an inductive approach was taken and semantic themes were developed. Firstly, data immersion and familiarization occurred, and preliminary observations were made. Then the data were systematically analyzed, and initial codes were assigned to every data item. Following this, themes were developed from the codes, capturing important patterns and experiences. Next, themes were reviewed and refined by checking them against the codes and the entire dataset until they accurately represented participants' comments and feedback. Lastly, themes were defined and named.

#### ERA Scale Modifications

Questions in Part C (the ERA Scale) with an ICC reliability level of poor or poor to moderate (<0.75) were analyzed and considered for rewording or removal. In addition, questions were amended based on participants' comments and feedback and an understanding of participants' user experience. Several new questions were created, relating to either topics with high ICC estimates from Part D [the Epilepsy Self-Management Scale (13)] or topics known to be important to risk (20) that had not been included in previous versions.

### Ethics Approval and Data Handling

Ethics approval was granted by NHS REC on 10/07/2015 (ID: 15/NW/0607). Research was conducted following Good Clinical Practice guidelines, and data were handled in accordance with the Data Protection Act 2018 (24). In line with this, participants' names were recorded on a sheet of paper with anonymized individual study participant numbers. These study participant numbers were used in all subsequent data handling. The sheet with participants' names and preferred contact details (phone number and/or email address) was kept in a locked cupboard in a locked room in a separate room of the hospital from where the data handling and analysis occurred. The research assistant was the only person with access to this sheet.

## Results

The test–retest of Part A (General Information), B [the Chalfont Seizure Severity Scale (14)], and C (the ERA Scale) of the questionnaire was completed by 102 participants, and 47 participants completed the test–retest of the optional Part D [the Epilepsy Self-Management Scale (13)]. The retest period spanned between 5 and 32 days, with the mean being 13.2 days (*SD* = 5.4). In total across both questionnaire completion phases, 24 questionnaires were completed in person at the outpatient clinic, 83 were completed over the phone, and 97 were completed via the online link.

Of the 102 participants, ages ranged from 18 to 81 years (*M* = 44), with 56 (55%) females and 46 (45%) males. The highest proportions of participants were single (*N* = 48, 47%) or married (*N* = 33, 32%). Most participants were White British (*N* = 52, 71%), followed by White Other (*N* = 10, 10%) and Black British (*N* = 8, 8%). Almost half were employed (*N* = 47, 46%), and the rest were not employed (*N* = 32, 31%), retired (*N* = 14, 14%), or other (*N* = 9, 9%). Educational attainment was university level for 42% of participants (*N* = 43), followed by other (*N* = 21, 21%), General Certificate of Secondary Education (GCSE) (*N* = 20, 20%), A-Level (*N* = 13, 13%), and prefer not to say (*N* = 5, 5%). Participants' responses to the demographic questions varied slightly in the test and retest phases; for example, some had their birthday in the period between, and their age therefore changed.

The number of antiepileptic medications that participants were prescribed ranged from 0 to 4+. The median number of medications prescribed was 2 (*N* = 30, 29%), and 1 medication was the mode (*N* = 34, 33%).

The highest proportions of participants said their seizures usually last between 1 and 10 min (*N* = 48, 47%) and 10 s to 1 min (*N* = 36, 35%); 82 (80%) participants lose awareness during seizures, and 58 (57%) have injured themselves during a seizure on at least one occasion.

### Intraclass Correlation Coefficient Estimates

As displayed in Table 1, across the 48 questions in Part C (the ERA Scale), ICC estimates ranged from 0.330 to 0.889. Based on the 95% confidence intervals of the ICC estimates, 17 (35.4%) of the questions have poor to moderate reliability levels, 4 (8.3%) have poor to good reliability levels, 19 (39.6%) have moderate to good reliability levels, 4 (8.3%) have good reliability levels, and 4 (8.3%) have good to excellent reliability levels.

**Table 1:** Intraclass correlation coefficient estimates and reliability levels for questions in Part C (the ERA Scale version 2).

**ERA Scale questions**	**ICC estimate**	**95% Confidence interval**		**ICC level of reliability**	***P*-value**
	**Mean of *k* measurements**	**Lower bound**	**Upperbound**		
**SECTION 1: PERSONAL SAFETY**					
1. Are people around you trained in first aid for seizures?	0.753	0.636	0.833	Moderate to good	<0.001
2. Do you have an individual emergency epilepsy plan (rectal diazepam or buccal midazolam or another antiepileptic medication)?	0.745	0.623	0.827	Moderate to good	<0.001
3. Do you have an up-to-date safety plan in place?	0.550	0.334	0.696	Poor to moderate	<0.001
4. Are people around you aware of the safety plan (e.g., which telephone number to call)?	0.568	0.362	0.708	Poor to moderate	<0.001
5. Is first aid equipment available and in good working order where necessary?	0.637	0.464	0.754	Poor to good	<0.001
6. Do you wear or carry any identification for your epilepsy (i.e., Medic Alert)?	0.860	0.792	0.905	Good to excellent	<0.001
7. Do you have a shower (heat controlled) installed?	0.542	0.326	0.689	Poor to moderate	<0.001
8. Do you have a gas cooker or halogen hob?	0.589	0.390	0.723	Poor to moderate	<0.001
9. Do your bathroom/toilet doors open outwards?	0.840	0.763	0.892	Good	<0.001
10. Is there an epilepsy alarm in your bedroom?	0.830	0.748	0.885	Good	<0.001
11. Do you have a fall alarm that is set off by a seizure?	0.609	0.420	0.736	Poor to moderate	<0.001
12. Are there side rails on your bed?	0.779	0.674	0.851	Moderate to good	<0.001
13. Are protective devices in good condition and regularly checked?	0.779	0.673	0.851	Moderate to good	<0.001
14. Are all your seizure related injuries noted and investigated?	0.760	0.646	0.838	Moderate to good	<0.001
**SECTION 2: HEALTH CARE**					
1. Are all your seizure events described in detail?	0.589	0.390	0.722	Poor to moderate	<0.001
2. Is your type of epilepsy or epileptic syndrome identified?	0.620	0.439	0.743	Poor to moderate	<0.001
3. Are all your seizures recorded in a seizure diary?	0.889	0.836	0.925	Good to excellent	<0.001
4. Do you have diagnostic tests regarding your epilepsy (EEG, CT scan, and MRI scan) when necessary?	0.445	0.179	0.625	Poor to moderate	0.002
5. Is the cause of your epilepsy known?	0.753	0.635	0.833	Moderate to good	<0.001
6. Do you think that your seizures are well controlled?	0.659	0.494	0.770	Poor to good	<0.001
7. Apart from your epilepsy, do you have any other medical problems?	0.797	0.700	0.863	Moderate to good	<0.001
8. Do you see a neurologist regarding your epilepsy and its management when your seizures are not well controlled or when you have significant drug side effects?	0.765	0.652	0.841	Moderate to good	<0.001
9. Do you go for appointments at a hospital outpatient clinic?	0.746	0.624	0.829	Moderate to good	<0.001
10. Do you visit your doctor (general practitioner)?	0.571	0.365	0.710	Poor to moderate	<0.001
11. Do you see a neurologist?	0.835	0.756	0.889	Good	<0.001
12. Do you see a specialist epilepsy nurse?	0.879	0.821	0.918	Good to excellent	<0.001
13. Do you see any other doctor or nurse?	0.330	0.006	0.549	Poor to moderate	0.023
14. Do you attend your appointments regularly?	0.772	0.662	0.846	Moderate to good	<0.001
15. Do you attend appointments when necessary?	0.505	0.271	0.664	Poor to moderate	<0.001
16. Are your antiepileptic drug levels measured regularly?	0.852	0.781	0.900	Good to excellent	<0.001
17. Do you go for blood tests [e.g., full blood count (FBC) of different types of blood cells, thyroid function tests (TFTs), and liver function test (LFT)]	0.534	0.311	0.685	Poor to moderate	<0.001
18. Are people around you able to tell when you are experiencing potential drug side effects and/or toxicity?	0.671	0.513	0.778	Moderate to good	<0.001
19. Are efforts made to look for any signs of drug side effects or toxicity when necessary?	0.626	0.446	0.748	Poor to moderate	<0.001
20. Is appropriate action taken if an adverse drug effect or toxicity is noticed?	0.497	0.255	0.660	Poor to moderate	<0.001
21. Are the drugs that you are prescribed collected from your pharmacy/chemist regularly?	0.812	0.722	0.873	Moderate to good	<0.001
22. Do you take your antiepileptic drugs as prescribed?	0.595	0.399	0.727	Poor to moderate	<0.001
23. Are you satisfied with the way in which your antiepileptic drugs are dispensed?	0.680	0.525	0.784	Moderate to good	<0.001
24. Is antiepileptic medication the only medicine you take?	0.796	0.699	0.862	Moderate to good	<0.001
25. Do you and the people who take you to health-care appointments have enough information to discuss your treatment plans?	0.383	0.088	0.582	Poor to moderate	0.008
**SECTION 3: QUALITY OF LIFE**					
1. Do your daily activities include a variety of preferred, interesting, and stimulating experiences?	0.679	0.525	0.783	Moderate to good	<0.001
2. Do you follow a healthy diet as recommended by your health practitioner?	0.647	0.476	0.762	Poor to good	<0.001
3. Are your sleep patterns regular and sufficient to avoid sleep deprivation?	0.698	0.554	0.796	Moderate to good	<0.001
4. Are your bowels regular and sufficient to avoid diarrhea or constipation?	0.668	0.510	0.776	Moderate to good	<0.001
5. Are you aware of what might set off your seizures?	0.838	0.759	0.891	Good	<0.001
6. Are you able to avoid these triggers of your epilepsy?	0.797	0.699	0.863	Moderate to good	<0.001
7. Do you think that your mood and behavior are taken into account in your treatment?	0.459	0.198	0.635	Poor to moderate	0.001
8. Do you have access to mental health care should you need it?	0.792	0.692	0.860	Moderate to good	<0.001
9. Do you have access to counseling should you need it?	0.661	0.499	0.771	Poor to good	<0.001

*EEG, electroencephalogram*.

For questions in Part D [the Epilepsy Self-Management Scale (13)], ICC estimates ranged from 0.312 to 0.943.

### Epilepsy Risk Awareness Scale (Version 2) Ratings

Participants were asked to rate Part C (the ERA Scale) as “A source of useful information” on a scale from 1 to 10. Participants' ratings ranged between 1 and 10, and the mean score was 7.4 (*N* = 101).

Participants were asked to rate Part C (the ERA Scale) as “Easy to understand and use” on a scale from 1 to 10. Participants' ratings ranged between 2 and 10, and the mean score was 8.2 (*N* = 102).

### Themes

Of the 102 participants, 43 (44%) provided 54 comments and feedback, which ranged from two words to multiple sentences and were both practical and emotional in nature. As the explained purpose of the research was to refine the ERA Scale, responses were predominantly suggestions for improvement. Four key themes were identified.

#### “Helpful” Tool

The notion of the ERA Scale as a “helpful” tool was expressed by multiple participants. Some found it helpful as it provided food for thought; the questions prompted them to evaluate their lives and their epilepsy in ways they may not have done before: “Made me think of things I'm not aware of and haven't thought about” (Patient [P]12). Some participants felt it was a useful source of information, as it showed people “what their entitled to” (P60), which is further evidenced by participants' positive ratings of the ERA Scale. Specifically, several participants noted the importance of the questions relating to mental health and/or counseling support: “Helpful in terms of suggestions for support e.g., mental health care—I'd like to know if there is help available and I'd like to be spoken to about that.” (P28). Others appreciated the interesting nature of the research and how it “Would be good for this research to continue” (P64), and how the outcome will be “helpful to people” with epilepsy (P112).

#### The Questionnaire Design Does Not Suit the Complexity of Epilepsy

The most prominent theme in this data, raised by many participants, is that the design of the questionnaire does not fully recognize and allow for the complexity of epilepsy. A main reason provided is that the response options are not sufficient and flexible enough as “Epilepsy is not black and white” (P76). Participants expressed it was often “difficult to narrow down to the multiple choice options available” (P52) as they have different types and strengths of seizures. Participants suggested it would be useful to include “sometimes” (P52; P40), “other” (P87), or “unsure” (P118) options to accommodate the variations in their epilepsy. Participants also felt there should be an option to expand on or “qualify an answer” (P10). As detailed by P76, “There should be an additional information box after each question to allow people to elaborate on their answers as each sufferer is different.”

#### Some Risk-Related Issues Are Missing

Many participants wrote about either their own or general risk-related issues that they felt were important to be emphasized or included in the questionnaire. Of these, the four issues that came up multiple times were as follows: memory (“there was nothing about memory loss after a seizure, yet that is one thing i suffer from hugely” P76); time of last seizure (“My last attack was over 5 years ago—this infrequency was influenced by my answers.” P7); lifestyle changes (“Include the affect of epilepsy on your lifestyle, any major events, any major decisions, any major changes over time.” P63); and mood (“Mood and treatment—it has been noted by people that I am much more short fused since I changed the medication, and it is actually a problem.” P52).

#### Therapeutic Benefits of Participating via Phone

Participants' enjoyment of, and the therapeutic benefits derived from, completing the ERA Scale over the phone with the research assistant was a prevalent theme. Multiple participants expressed appreciation for being able to share their lived experiences in this way: “… you've given me time on the phone to explain what's happening and this is the first time that's happened in 22 years—thank you” (P54), and specifically to “speak to someone about my experience 1-1” (P100). These participants emphasized that it was “very nice to talk with someone who is understanding and helps me understand” (P39), and this led one individual to comment “Wish there was someone to ring up once every 2 months to check in and say ‘how's it going?' and check in about the questionnaire answers” (P54). What has been highlighted through these participants' comments is the added value of feeling cared for while completing this questionnaire about their risk level: “I really appreciate you listening to me and showing you care” (P11).

### Final Test of the Epilepsy Risk Awareness Scale (Version 3)

Of the original 102 participants, 32 completed the amended ERA Scale (version 3) via an online link. The new “sometimes” response option was selected for 17% of answers, and unlike in the test–retest phases, participants did not write any suggestions for the improvement of the questionnaire design or content in the optional comments box. The mean time taken to complete the ERA Scale (version 3) was 7 min, the mode time was 5 min, and a substantial reduction from 20 min for the draft questionnaire.

The ERA Scale (version 3) is now composed of two parts: (A) which consists of 9 questions and (B) which consists of 40 questions across four sections—Your Epilepsy, Your Personal Safety, Your Physical Wellbeing, and Your Mental Wellbeing (see in Supplementary Materials). Response options are “yes,” “sometimes,” “no,” and “not applicable.”

## Discussion

The ERA Scale is the only risk assessment tool, to our knowledge, developed to be used by both people with epilepsy and health-care professionals, that approaches risk holistically and aims to improve quality of life. The present research refined the ERA Scale based on test–retest reliability of questions and feedback from participants. Based on our findings, questions were amended, removed, or added to the ERA Scale, in addition to other modifications. Patient feedback highlighted that the response options were not sufficient to accommodate the variability in living with epilepsy, so they were expanded to include a “sometimes” option. In the final test phase, “sometimes” was selected in 17% of answers, so the expanded response options should enable patients to provide more accurate answers. There remains an optional comments box at the end, as it is understood that users may want to expand upon their answers (25). Additionally, as the Quality of Life section of the ERA Scale did not reach a high level of internal consistency in the previous research phase (18) and new questions were added in the present research, it was broken down into two new sections—Your Physical Wellbeing and Your Mental Wellbeing. This was to support ease of answering, as questions are grouped based on the same underlying constructs (26). Lastly, in order to reduce the response burden, which can be defined as “the effort required by the patient to answer a questionnaire,” (27) and to improve practicality, the ERA Scale was reduced in length to take 7 min on average. These changes resulted in the final ERA Scale (version 3, see Supplementary Material).

This research supports the importance of engaging with patients with the chronic condition of epilepsy in a way that extends beyond their medical symptoms. Chronic conditions pervade everyday life, so risk can come in many different forms—for example, medical, environmental, social, and psychological. The ERA Scale could encourage conversations that enable health-care professionals to understand patients on a more personal, holistic level. Ha and Longnecker's (2010) research (28) suggests that these more meaningful interactions enhance patient–clinician relationships, improving health outcomes and overall patient engagement. These relationships are particularly important for chronic lifestyle-related conditions as patients often prefer to have a long working relationship with a clinician who understands their condition in the context of their lives (29). In addition to having the potential to support relationship building, the ERA Scale also provides a structure for consultations, drawing a parallel with the Calgary-Cambridge model (30). This can assist up-front agenda setting for both patients and health-care professionals, which has been associated with multiple positive outcomes, including improving patients' evaluations of the quality of the interaction, improving clinicians' understanding of patients' concerns, and reducing the number of unmet patient concerns (31). The ERA Scale can also be used to standardize clinical consultation notes, improving documentation and ultimately patient care, (32) a need that initially led to the development of the ERA Scale.

The simple act of being listened to has powerful therapeutic benefits in health care (33), and this was raised in the present research by participants in their comments and feedback. It is through listening that a health-care professional acknowledges the importance of a patient's voice (34). This is central to the paradigm shift from paternalism to patient-centered care (35). In this mutual participation model (36), patients are listened to, viewed as experts in their own life experiences and conditions, and considered equal partners with clinicians. This has been linked to improved clinical outcomes, self-management and compliance with treatment plans (37), enhanced patient satisfaction and increased disclosure of problems and emotions (38). People with epilepsy and other chronic conditions are already in charge of their own health on a day-to-day basis, so the ERA Scale provides a template for them to share their wider experiences and for health-care professionals to listen to them, encouraging shared decision making (39).

A limitation of this research is the variability during the test–retest phase, as the retest period spanned from 5 to 32 days, and participants completed the ERA Scale in different ways—in person, over the phone, or via an online link. In addition, several questions that have been included in the final version of the ERA Scale have poor to moderate ICC reliability levels, although the wording has been improved, and several new questions have not been assessed for test–retest reliability. However, these questions are key to the management of epilepsy and the low test–retest reliability reflects the dynamic and changeable nature of epilepsy. Despite these limitations, the sample size is a key strength, as it far exceeds the minimum requirements to detect the value of the ICC (22, 40). In addition, the lack of participant exclusion criteria ensured the sample was as representative as possible within the demographic of the area of the Royal Free Hospital. Of the 102 participants, 7(8%) had an intellectual disability, compared with an estimated 20% of the UK population with epilepsy and an intellectual disability (41). Overall, the ERA Scale has been validated in multiple phases, in line with the questionnaire validation guidelines outlined by Tsang et al. (42), and the methodology has ensured that patients are involved throughout.

Our research group is developing a mobile app to host the ERA Scale, which will enable it to be used as a quantified scale with question weightings and subsequent risk scores. This aligns with the wider transition to e-health care and an understanding of how it can revolutionize chronic disease management (43) and improve quality of life for people with epilepsy. As an app, the ERA Scale also provides further opportunities for both quantitative and qualitative data collection and research, to improve knowledge and clinical practice; and it will increase its accessibility for people with epilepsy to use independently. We intend to incorporate the ERA Scale into care plans, in particular for people with uncontrolled epilepsy and comorbidities such as depression and intellectual disabilities. Future research on the ERA Scale will examine its utility as a tool to assess risk over a 12-month period for people with epilepsy in a variety of populations, and whether its use translates to improved outcomes.

## Conclusions

The ERA Scale (version 3) embodies the essence of patient-centered care; it facilitates a holistic, tailored, and structured conversation between people with epilepsy and their health-care professionals, and it encourages shared decision making (39) to alter risk-related strategies to improve quality of life.

## Data Availability Statement

The datasets generated for this study are available on request to the corresponding author.

## Ethics Statement

The studies involving human participants were reviewed and approved by NHS Research Ethics Committee on 10/07/2015 (ID:15/NW/0607). The patients/participants provided their informed consent to participate in this study.

## Author Contributions

HA-L and CC originated the idea for the work and developed earlier versions of the ERA Scale. RI recruited participants, collected the data, conducted the data analysis, and drafted the manuscript. RI, HA-L, and VK amended the ERA Scale. VK created the online version of the ERA Scale (version 2) that was used in this research. All authors reviewed and commented on the manuscript and approved it for publication.

## Conflict of Interest

The authors declare that the research was conducted in the absence of any commercial or financial relationships that could be construed as a potential conflict of interest.
