# Supplementary material for: The Epilepsy Risk Awareness (ERA) Scale: A New Era for Holistic Risk Assessment in Epilepsy
Source: Front Neurol. 2020 Jun 11;11:465. doi: 10.3389/fneur.2020.00465 (PMC7300212; doi:10.3389/fneur.2020.00465)
Supplement: Supplementary file 1 [file Data_Sheet_1.PDF]

Epilepsy Risk Awareness scale

Appendix: ERA scale

Quantification and Piloting of an Epilepsy Risk Awareness scale

Background:

About one in 20 people will have some form of seizure or epilepsy during their life, so it is a common and important condition. We need to balance protecting people with epilepsy from risks while avoiding unnecessary restrictions. To help get this balance right, we have developed the Epilepsy Risk Awareness Checklist (ERAC), summarising a person's individual profile of Personal Safety (Section 1), Health Care (Section 2) and Quality of Life (Section 3). We now wish to test out the Checklist with a group of epilepsy patients so see how useful it might be in practice. If you have a carer, we are happy for them to help you in completing this questionnaire (with your agreement).

Instructions:

This questionnaire consists of four parts: A - General information about yourself, B - the Epilepsy Severity Scale, C - the Epilepsy Risk Awareness Checklist and D – the Self-Management Scale. Please tick your answers in the appropriate boxes. Space is provided for any comments that you might have about particular questions. After we have received the completed questionnaires we will analyse the results and design a modified ERA Scale.

Before you start, please tick to confirm that you have signed and returned the consent form: ☐

Part A – Please tell us a little about yourself.

1. **Age:** ___________ Prefer not to say☐
2. **Gender:** Female☐ Male☐ Transgender☐ Prefer not to say☐
3. **Marital Status:** Married☐ Widowed☐ Divorced☐ Single☐ Partnership☐ Separated☐ Prefer not to say☐
4. **Religion:** None☐ Christian☐ Muslim☐ Hindu☐ Jewish☐ Other☐________________ Prefer not to say☐
5. **Ethnicity:** White British☐ White Other☐ Black British☐ Black Other☐ Asian British☐ Asian Other ☐ Other☐_______________ Prefer not to say☐
6. **Current employment:** Employed☐ Not Employed☐ Retired☐ Other☐ _____________
7. **Education:** University☐ A-Level☐ GCSE☐ Other☐ __________ Prefer not to say☐
8. **Number of anti-epileptic medicines that you are currently prescribed**:

   0☐ 1☐ 2☐ 3☐ 4+☐
9. **Postcode for where you live:** _________________
10. **Study participant number as sent to you by email:** ______________

University of Hertfordshire UH | Royal Free London NHS Foundation Trust | Central London Community Healthcare NHS Trust

© Braun, Kendall, Cole, Smeeton, Angus-Leppan 2016

Part B – Seizure Severity Scale*

Please describe what usually happens during one of your seizures.

***Duncan JS, Sander JW. The Chalfont Seizure Severity Scale. JNNP. 1991;54(10):873-6.**

TOTAL = 178

Part C – Epilepsy Risk Awareness Checklist

This is the main part of this questionnaire. Please tick all questions.

ERAC Section 1: Personal Safety

1. *Are people around you trained in first aid for seizures?* (4)

   Yes☐ No☐ Comments: ……………………………………………………………………...

   Not Applicable☐ ………………………………………………………………………

2. *Do you have an individual emergency epilepsy plan (rectal diazepam or buccal midazolam* or another antiepileptic medication)? (1)

   Yes☐ No☐ Comments: ……………………………………………………………………...

   Not Applicable☐ ………………………………………………………………………

3. *Do you have an up to date safety plan in place?* (1)

   Yes☐ No☐ Comments: ……………………………………………………………………...

   Not Applicable☐ ………………………………………………………………………

4. *Are people around you aware of the safety plan (e.g. which telephone number to call)* (2)

   Yes☐ No☐ Comments: ……………………………………………………………………...

   Not Applicable☐ ………………………………………………………………………

5. *Is first aid equipment available and in good working order where necessary?* (2)

   Yes☐ No☐ Comments: ……………………………………………………………………...

   Not Applicable☐ ………………………………………………………………………

6. *Do you wear or carry any identification for your epilepsy (i.e. Medic Alert)?* (1)

   Yes☐ No☐ Comments: ……………………………………………………………………...

   Not Applicable☐ ………………………………………………………………………

7. *Do you have a shower (heat controlled) installed?* (2)

   Yes☐ No☐ Comments: ……………………………………………………………………...

   Not Applicable☐ ………………………………………………………………………

© Braun, Kendall, Cole, Smeeton, Angus-Leppan 2016

8. *Do you have a gas cooker or halogen hob?* (1)

   Yes☐ No☐ Comments: ………………………………………………………………………....

   Not Applicable☐ ……………………………………………………………………………

9. *Do your bathroom/ toilet doors open outwards?* (1)

   Yes☐ No☐ Comments: ………………………………………………………………………....

   Not Applicable☐ ……………………………………………………………………………

10. *Is there an epilepsy alarm in your bedroom?* (1)

   Yes☐ No☐ Comments: ………………………………………………………………………....

   Not Applicable☐ ……………………………………………………………………………

11. *Do you have a fall alarm that is set off by a seizure?* (1)

   Yes☐ No☐ Comments: ………………………………………………………………………....

   Not Applicable☐ ……………………………………………………………………………

12. *Are there side rails on your bed?* (1)

   Yes☐ No☐ Comments: ………………………………………………………………………....

   Not Applicable☐ ……………………………………………………………………………

13. *Are protective devices in good condition and regularly checked?* (2)

   Yes☐ No☐ Comments: ………………………………………………………………………....

   Not Applicable☐ ……………………………………………………………………………

14. *Are all your seizure related injuries noted and investigated?* (3)

   Yes☐ No☐ Comments: ………………………………………………………………………....

   Not Applicable☐ ……………………………………………………………………………

ERAC - Section 2: Health Care

1. *Are all your seizure events described in detail?* (4)

   Yes☐ No☐ Comments: ………………………………………………………………………....

   Not Applicable☐ ……………………………………………………………………………

2. *Is your type of epilepsy or epileptic syndrome identified?* (2)

   Yes☐ No☐ Comments: ………………………………………………………………………....

   Not Applicable☐ ……………………………………………………………………………

3. *Are all your seizures recorded in a seizure diary?* (3)

   Yes☐ No☐ Comments: ………………………………………………………………………....

© Braun, Kendall, Cole, Smeeton, Angus-Leppan 2016

Not Applicable☐ ……………………………………………………………………………

4. *Do you have diagnostic tests regarding your epilepsy (EEG, CT, MRI) when necessary?* (2)

Yes☐ No☐ Comments: …………………………………………………………………....

Not Applicable☐ ……………………………………………………………………………

5. *Is the cause of your epilepsy known?* (1)

Yes☐ No☐ Comments: …………………………………………………………………....

Not Applicable☐ ……………………………………………………………………………

6. *Do you think that your seizures are well controlled?* (4)

Yes☐ No☐ Comments: …………………………………………………………………....

Not Applicable☐ ……………………………………………………………………………

7. *Apart from your epilepsy, do you have any other medical problems?* (3)

Yes☐ No☐ Comments: …………………………………………………………………....

Not Applicable☐ ……………………………………………………………………………

8. *Do you see a neurologist regarding your epilepsy and its management when your seizures are not well controlled or when you have significant drug side effects?* (4)

Yes☐ No☐ Comments: …………………………………………………………………....

Not Applicable☐ ……………………………………………………………………………

9. *Do you go for appointments at a hospital outpatient clinic?* (3)

Yes☐ No☐ Comments: …………………………………………………………………....

Not Applicable☐ ……………………………………………………………………………

10. *Do you visit your doctor (general practitioner)?* (3)

Yes☐ No☐ Comments: …………………………………………………………………....

Not Applicable☐ ……………………………………………………………………………

11. *Do you see a neurologist?* (3)

Yes☐ No☐ Comments: …………………………………………………………………....

Not Applicable☐ ……………………………………………………………………………

12. *Do you see a specialist epilepsy nurse?* (3)

Yes☐ No☐ Comments: …………………………………………………………………....

Not Applicable☐ ……………………………………………………………………………

University of Hertfordshire UH Royal Free London NHS NHS Foundation Trust Central London NHS Community Healthcare NHS Trust

© Braun, Kendall, Cole, Smeeton, Angus-Leppan 2016

13. *Do you see any other doctor or nurse?* (1)

Yes☐ No☐ Comments: ……………………………………………………………………………..

Not Applicable☐ ……………………………………………………………………………

14. *Do you attend your appointments regularly?* (2)

Yes☐ No☐ Comments: ……………………………………………………………………………..

Not Applicable☐ ……………………………………………………………………………

15. *Do you attend appointments when necessary?* (3)

Yes☐ No☐ Comments: ……………………………………………………………………………..

Not Applicable☐ ……………………………………………………………………………

16. *Are your antiepileptic drug levels measured regularly?* (2)

Yes☐ No☐ Comments: ……………………………………………………………………………..

Not Applicable☐ ……………………………………………………………………………

17. *Do you go for blood tests?* (e.g. FBC – Full Blood Count of different types of blood cells. TFT – Thyroid Function Tests and LFT – Liver Function Test) (2)

Yes☐ No☐ Comments: ……………………………………………………………………………..

Not Applicable☐ ……………………………………………………………………………

18. *Are people around you able to tell when you are experiencing potential drug side effects and/or toxicity?* (4)

Yes☐ No☐ Comments: ……………………………………………………………………………..

Not Applicable☐ ……………………………………………………………………………

19. *Are efforts made to look for any signs of drug side effects or toxicity when necessary?* (4)

Yes☐ No☐ Comments: ……………………………………………………………………………..

Not Applicable☐ ……………………………………………………………………………

20. *Is appropriate action taken if an adverse drug effect or toxicity is noticed?* (4)

Yes☐ No☐ Comments: ……………………………………………………………………………..

Not Applicable☐ ……………………………………………………………………………

21. *Are the drugs that you are prescribed collected from your pharmacy/chemist regularly?* (3)

Yes☐ No☐ Comments: ……………………………………………………………………………..

Not Applicable☐ ……………………………………………………………………………

22. *Do you take your antiepileptic drugs as prescribed?* (4)

Yes☐ No☐ Comments: ……………………………………………………………………………..

Not Applicable☐ ……………………………………………………………………………

University of Hertfordshire UH | Royal Free London NHS Foundation Trust | Central London Community Healthcare NHS Trust

© Braun, Kendall, Cole, Smeeton, Angus-Leppan 2016

23. *Are you satisfied with the way in which your antiepileptic drugs are dispensed?* (2)

Yes☐ No☐ Comments: …………………………………………………………………….....

Not Applicable☐ ………………………………………………………………………

24. *Is antiepileptic medication the only medicine that you take?* (4)

Yes☐ No☐ Comments: …………………………………………………………………….....

Not Applicable☐ ………………………………………………………………………

25. *Do you and the people who take you to healthcare appointments have enough information to discuss your treatment plans?* (4)

Yes☐ No☐ Comments: …………………………………………………………………….....

Not Applicable☐ ………………………………………………………………………

Part C - Section 3: Quality of Life

1. Do your daily activities include a variety of preferred, interesting and stimulating experiences? (1)

Yes☐ No☐ Comments: …………………………………………………………………….....

Not Applicable☐ ………………………………………………………………………

2. Do you follow a healthy diet as recommended by your health practitioner? (2)

Yes☐ No☐ Comments: …………………………………………………………………….....

Not Applicable☐ ………………………………………………………………………

3. Are your sleep patterns regular and sufficient to avoid sleep deprivation? (4)

Yes☐ No☐ Comments: …………………………………………………………………….....

Not Applicable☐ ………………………………………………………………………

4. Are your bowels regular and sufficient to avoid diarrhoea or constipation? (2)

Yes☐ No☐ Comments: …………………………………………………………………….....

Not Applicable☐ ………………………………………………………………………

5. Are you aware of what might set off your seizures? (4)

Yes☐ No☐ Comments: …………………………………………………………………….....

Not Applicable☐ ………………………………………………………………………

6. Are you able to avoid these triggers of your epilepsy? (3)

Yes☐ No☐ Comments: …………………………………………………………………….....

Not Applicable☐ ………………………………………………………………………

© Braun, Kendall, Cole, Smeeton, Angus-Leppan 2016

7. Do you think that your mood and behaviour are taken into account in your treatment? (4)

   Yes☐ No☐ Comments: …………………………………………………………………………....

   Not Applicable☐ ……………………………………………………………………………

8. Do you have access to mental health care should you need it? (4)

   Yes☐ No☐ Comments: …………………………………………………………………………....

   Not Applicable☐ ……………………………………………………………………………

9. Do you have access to counselling should you need it? (3)

   Yes☐ No☐ Comments: …………………………………………………………………………....

   Not Applicable☐ ……………………………………………………………………………

TOTAL = 124

How would you rate the **Epilepsy Risk Awareness Checklist** (**Part C of this questionnaire)?**
Use the scale below of 1-10 with 1 = very poor and 10 = excellent in terms of being:

A useful source of information:	1	2	3	4	5	6	7	8	9	10
Easy to understand and use:	1	2	3	4	5	6	7	8	9	10

Part D – Epilepsy Self-Management Scale*

Instructions: The following statements describe what people do to manage their epilepsy. Please circle one number for each statement to show how often you do the following. As you answer the questions, please think about your activities in the past year.

Dilorio C. Epilepsy Self-Management Scale 2010 [Available from: http://managingepilepsywell.org/documents/instruments/Epilepsy%20Self%20Mangement-Scale%20and%20Description.pdf.]

TOTAL = 190

© Braun, Kendall, Cole, Smeeton, Angus-Leppan 2016
