# Supplementary material for: The Epilepsy Risk Awareness (ERA) Scale: A New Era for Holistic Risk Assessment in Epilepsy
Source: Front Neurol. 2020 Jun 11;11:465. doi: 10.3389/fneur.2020.00465 (PMC7300212; doi:10.3389/fneur.2020.00465)
Supplement: Supplementary file 2 [file Data_Sheet_2.pdf]

EPILEPSY RISK AWARENESS (ERA) SCALE

(VERSION 3)

BACKGROUND
Approximately 50 million people worldwide have epilepsy, and others can have a one-off seizure at some point in their lives. The ERA Scale is a tool that measures an individual's risk, looking holistically at multiple areas of their life that may be affected by epilepsy. The ERA Scale aims to improve quality of life by balancing protection from risks and avoiding undue restrictions.

INSTRUCTIONS
This questionnaire consists of two parts: Part A) General Information, and Part B) the ERA Scale, which has four sections: Your Epilepsy, Your Personal Safety, Your Physical Wellbeing, and Your Mental Wellbeing.

Please answer EVERY question by selecting the option most relevant to you with a "X". You can either complete the questionnaire by yourself or with the support of others, such as carers, family members and health care professionals.

NHS/Reference Number: ______________________ **Today's Date:** ______________________

PART A

1. Age: ______________________ Prefer not to say ☐

2. Gender: Female ☐ Male ☐ Transgender ☐ Other ☐ Prefer not to say ☐

3. Ethnicity: White British ☐ White Other ☐ Black British ☐ Black Other ☐ Asian British ☐ Asian Other ☐ Other ☐ Prefer not to say ☐

4. Marital status: Single ☐ Married ☐ Partnership ☐ Divorced ☐ Widowed ☐ Other ☐ Prefer not to say ☐

5. Current employment status: Employed ☐ Not employed ☐ Retired ☐ Unable to work due to epilepsy ☐ Other ☐ Prefer not to say ☐

6. Educational background: University ☐ A-Level ☐ GCSE ☐ Other ☐ Prefer not to say ☐

7. Do you currently drive a car? Yes ☐ No ☐

8. How many antiepileptic medications are you prescribed at the moment?

0 ☐ 1 ☐ 2 ☐ 3 ☐ 4+ ☐

9. How long ago did you have your last seizure? Today ☐ This week ☐ This month ☐ This year ☐ More than 1 year ago ☐ More than 2 years ago ☐ More than 5 years ago ☐

© Angus-Leppan, Ison, Kisan, Braun, Kendall, Smeeton, Cole, 2020.

PART B

SECTION 1: YOUR EPILEPSY	YES	SOMETIMES	NO	NOT APPLICABLE
1. Do you keep a record of your seizures?				
2. Do you think that your seizures are well controlled?				
3. Are you aware of what might set off your seizures?				
4. Are you able to avoid the triggers of your epilepsy?				
5. Do you lose awareness during a seizure?				
6. Do you fall to the ground during a seizure?				
7. Is your type of epilepsy or epileptic syndrome identified?				
8. Is the cause of your epilepsy known?				
9. Are you and/or those around you able to tell when you are experiencing potential medication side effects or toxicity?				
10. Do you contact your neurology team when your seizures are not well controlled and/or when you have significant medication side effects?				
11. Do you go for appointments at a specialist outpatient clinic for your epilepsy (e.g. to see a neurologist or specialist epilepsy nurse)?				
12. Do you visit your general practitioner (GP) regarding your epilepsy?				
13. Do you and/or your carer collect your prescribed medication from your pharmacist/chemist regularly?				
14. Do you take your antiepileptic medication as prescribed?				
15. Do you have a way to remind yourself to take your medication (e.g. alarm on your phone, dosette box, family/friend/carer)?				
16. Is antiepileptic medication the only medication you take?				
17. Apart from your epilepsy, do you have any other medical problems?				
18. Do you and/or the people who take you to healthcare appointments have enough information to discuss your treatment plans?				
**SECTION 2: YOUR PERSONAL SAFETY**	**YES**	**SOMETIMES**	**NO**	**NOT APPLICABLE**
19. Do you wear or carry any identification for your epilepsy (e.g. Medic Alert)?				
20. Are people around you trained in first aid for seizures?				
21. Do you have an individual emergency epilepsy plan (e.g. rectal diazepam, buccal midazolam, another antiepileptic medication)?				
22. Do you live alone?				
23. Do you sleep alone?				
24. Do you ever have a bath alone?				
25. Do your bathroom/toilet doors open outwards?				

© Angus-Leppan, Ison, Kisan, Braun, Kendall, Smeeton, Cole, 2020.

26. Do you have an epilepsy alarm (e.g. in your bedroom, a fall alarm)?				
27. Are your protective devices (e.g. alarms, side rails) in good condition and checked regularly?				
28. Are all of your seizure related injuries noted and investigated?				
**SECTION 3: YOUR PHYSICAL WELLBEING**	**YES**	**SOMETIMES**	**NO**	**NOT APPLICABLE**
29. Do you follow a healthy diet as recommended by your health practitioner?				
30. Have you or has anyone close to you expressed concerns about how much alcohol you drink?				
31. Do you exercise regularly?				
32. Are your sleep patterns regular and sufficient to avoid sleep deprivation?				
33. Do you have a satisfactory sex life?				
34. Do you have memory problems?				
**SECTION 4: YOUR MENTAL WELLBEING**	**YES**	**SOMETIMES**	**NO**	**NOT APPLICABLE**
35. Do your daily activities include a variety of interesting and stimulating experiences?				
36. Do you use healthy strategies to manage stress (e.g. listening to music, exercising, meditating)?				
37. Do you think that your mood is taken into account in your epilepsy treatment?				
38. Do you feel you need access to mental health care (e.g. counselling)?				
39. Do you talk with other people who have epilepsy?				
40. Do you have a strong support system (e.g. family, friends, neighbours, colleagues)?				

Optional: Please provide any comments or feedback below.

© Angus-Leppan, Ison, Kisan, Braun, Kendall, Smeeton, Cole, 2020.
